# Transition of an Anaerobic *Escherichia coli* Culture to Aerobiosis: Balancing mRNA and Protein Levels in a Demand-Directed Dynamic Flux Balance Analysis

**DOI:** 10.1371/journal.pone.0158711

**Published:** 2016-07-06

**Authors:** Joachim von Wulffen, RecogNice-Team, Oliver Sawodny, Ronny Feuer

**Affiliations:** Institute for System Dynamics, University of Stuttgart, Stuttgart, Germany; Hospital for Sick Children, CANADA

## Abstract

The facultative anaerobic bacterium *Escherichia coli* is frequently forced to adapt to changing environmental conditions. One important determinant for metabolism is the availability of oxygen allowing a more efficient metabolism. Especially in large scale bioreactors, the distribution of oxygen is inhomogeneous and individual cells encounter frequent changes. This might contribute to observed yield losses during process upscaling. Short-term gene expression data exist of an anaerobic *E*. *coli* batch culture shifting to aerobic conditions. The data reveal temporary upregulation of genes that are less efficient in terms of energy conservation than the genes predicted by conventional flux balance analyses. In this study, we provide evidence for a positive correlation between metabolic fluxes and gene expression. We then hypothesize that the more efficient enzymes are limited by their low expression, restricting flux through their reactions. We define a demand that triggers expression of the demanded enzymes that we explicitly include in our model. With these features we propose a method, demand-directed dynamic flux balance analysis, dddFBA, bringing together elements of several previously published methods. The introduction of additional flux constraints proportional to gene expression provoke a temporary demand for less efficient enzymes, which is in agreement with the transient upregulation of these genes observed in the data. In the proposed approach, the applied objective function of growth rate maximization together with the introduced constraints triggers expression of metabolically less efficient genes. This finding is one possible explanation for the yield losses observed in large scale bacterial cultivations where steady oxygen supply cannot be warranted.

## Introduction

The model organism *Escherichia coli* is a facultative anaerobic bacterium, i.e. it is able to grow in both aerobic and anaerobic environments. To do so, cells need to be able to adapt to changes of the growth conditions. This capability is required in both the natural habitat and in biotechnological applications where, due to inefficient mixing in large scale bioreactors, oxygen supply is unsteady [1–3]. Adaptation takes place on the transcriptional level in multiple ways, e.g. increased expression of genes for aerobic respiration, or decreased expression of less efficient fermentative genes. Due to the high reduction potential of molecular oxygen, cells are able to generate more energy from its substrates, e.g. sugars, in aerobic compared to anaerobic metabolism. Survival is hampered, however, by the toxic effects of oxygen. Oxygen can get reduced to form the superoxide anion ($O_{2}^{-}$) and other ROS which provoke oxidative damage to DNA, RNA, proteins, and lipids (reviewed in [4]).

Adaptation from anaerobic to aerobic metabolism requires extensive adjustments of the enzyme composition of the cell (more than 20% of the genome being differentially expressed [5]). In this article we will focus on the adjustment of metabolic enzyme expression. Cells are constantly competing against each other for resources and so the fastest growing and best adapted cells will prevail. On the other hand, gene expression is a costly and time-consuming process so cells need to evolve a way of minimizing the total adjustments required for adaptation [6]. Transient overexpression of enzymes, especially enzymes of the first steps of a pathway, allows maximal flux through the pathway with concurrent minimal adjustments of expression [7]. Transition data of transcript expression of a shift from anaerobic to aerobic conditions in an *E*. *coli* culture also indicate transient overexpression of multiple genes [5]. Furthermore, these data suggest expression of metabolically less efficient genes.

Lewis *et al*. [8] used flux balance analysis (FBA) to classify genes with respect to their essentiality and relative efficiency. In FBA, fluxes of the quasi-steady state metabolism of a culture are estimated by an optimization procedure [9]. FBA has been successful in predicting phenotypes of knock-out mutant strains, metabolite exchange rates and growth rates under different environmental conditions and in different organisms [10–13]. The flux distribution is modeled assuming a quasi-steady state of internal metabolites that are interconnected via reactions that are stored in the stoichiometric matrix [11, 12, 14]. The solution space is further constrained thermodynamically by forcing irreversible fluxes to be positive. Concerning the objective function of the optimization there are different approaches according to prerequisites and the addressed problem [13]. A frequently and successfully applied objective function for batch cultivations is maximization of the growth rate with concurrent restriction of substrate uptake (reviewed in [15]). The result of an FBA is then a growth optimal flux distribution of a quasi-steady state culture in the exponential growth phase.

In parsimonious FBA (pFBA) [8], this optimization is followed by a minimization of all fluxes within the solution space of the the growth optimum, to minimize the requirement for enzyme expression. Using this technique, genes can be classified according to whether it is essential, required for optimal growth, metabolically less efficient (MLE, i.e. less energy production, or more consumption when this gene is used), enzymatically less efficient (ELE, i.e. overall flux is higher when this gene is used), or whether the gene is not used in any case.

**Fig 1:**
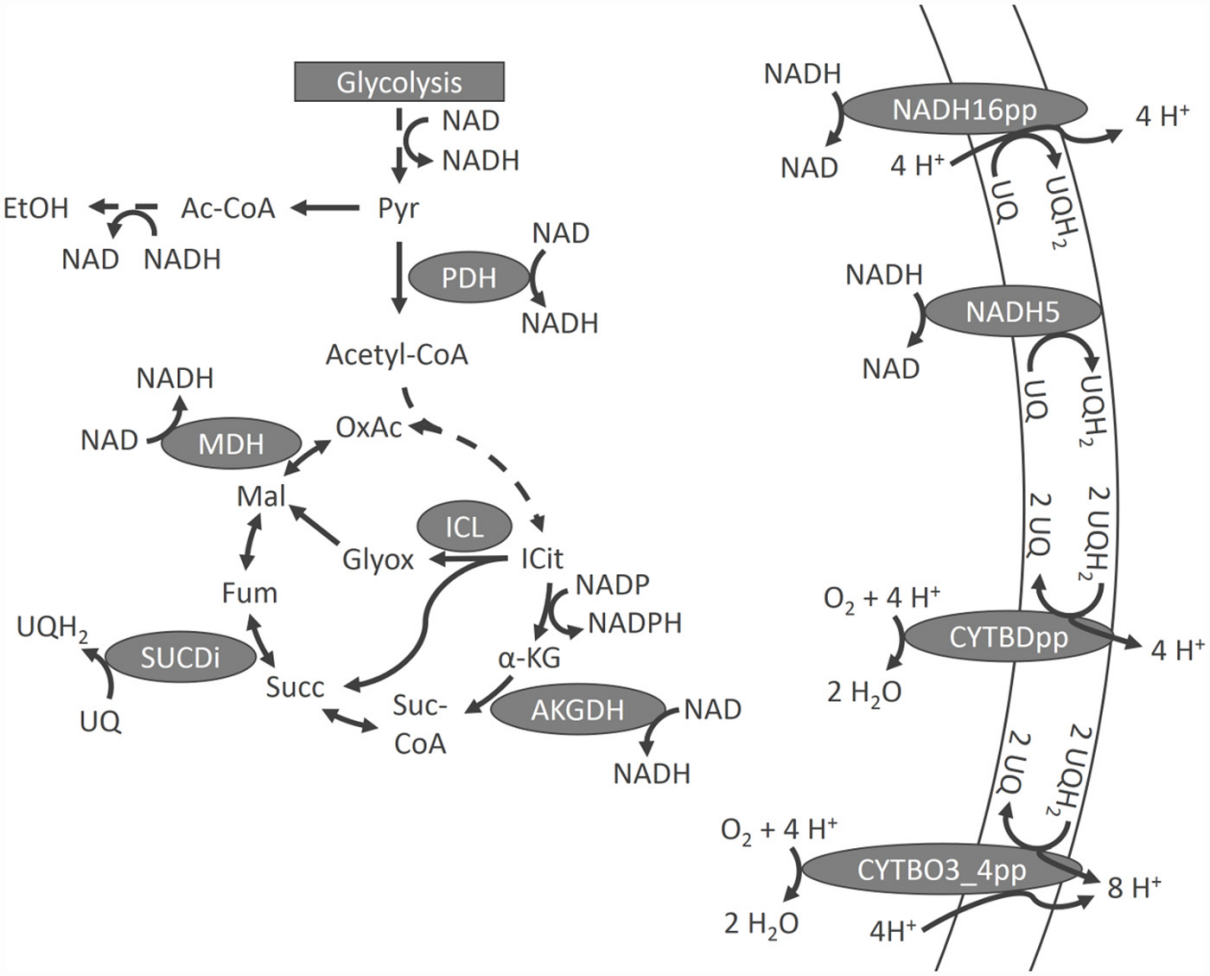
Outline of central aerobic metabolism. Reaction nomenclature according to iJO1366 [14]. Pyr, pyruvate; ICit, Isocitric acid; *α*-KG, *α*-ketoglutaric acid; Suc-CoA, succinyl-coenzyme A; Succ, succinic acid; Mal, malic acid; OxAc, oxaloacetic acid; UQ, ubiquinone; UQH_2_, ubiquinol; only redox cofactors are considered.

Transient upregulation of MLE genes might arise from limited capacity of the optimal enzymes. E.g., in the electron transport chain (ETC) the MLE gene *ndh* (catalyzing the reaction NADH5, reaction naming according to iJO1366 [14]) is transiently upregulated whereas expression of the optimal enzyme, encoded by the *nuo* operon (catalyzing the reaction NADH16pp), increases only slightly [5]. NADH5 is MLE, because it translocates no protons across the plasma membrane which could otherwise be used for energy conservation via ATPase (Fig 1). Similarly, expression of the *cyo* operon (catalyzing CYTBO3_4pp) increases after transition, whereas the MLE operon *cyd* (catalyzing CYTBDpp, Fig 1) is transiently overexpressed. Temporary overexpression is useful to minimize the required adjustments of gene expression [7], yet, this was only shown for essential pathways and not for MLE genes. If a transiently high flux through an optimal enzyme is beneficial for efficient growth, but the enzyme capacity is constrained due to underexpression, MLE genes might help overcome this shortage at the expense of optimal efficiency.

Dynamic FBA (dFBA) is an extension of FBA with the aim of simulating time course experiments [16]. In dFBA with static optimization approach (SOA) the simulation time is divided into small periods which are assumed to be in quasi-steady state. For every time step, an FBA problem is solved and the fluxes are integrated over the time period and extracellular concentrations are calculated, accordingly. dFBA was originally applied to simulations of diauxic growth and has been extended, e.g. with regulatory constraints [17–20].

These studies have in common that they are passive in terms of their regulation. A Boolean regulatory model is superimposed on the reaction network by addition of flux constraints that are deduced from the environmental state of the cell. Boolean models of gene regulation generally have the problem that the interplay between the factors is highly complex, therefore it is hard to identify and model the contribution of individual factors satisfactorily.

Several methods have been published that aim to improve flux predictions based on transcriptomic data. However, in an extensive comparison, none of the methods improved the overall outcome (determined by deviation from fluxes measured using ^13^C-labeled substrates) compared to pFBA which does not incorporate transcriptomic data [21]. This indicates that for most cases the constraints introduced prevent the FBA from correctly identifying the parsimonious optimum which better approximates the true flux distribution than the other methods. We therefore decided not to use transcription data directly to constrain the metabolic model (as e.g. in [22–25]), but to simulate mRNA and protein expression explicitly and then deduce constraints depending on these simulated values. Since flux measurement data are only available for steady states, they cannot be used as a benchmark here. We therefore investigate whether the principle behavior of gene expression matches our simulations with the enzymatic capacity constraints and objective functions suggested.

## Results and Discussion

### Correlation of transcriptome data with FBA fluxes

To investigate whether gene expression can serve as an indicator for respective flux rates, we analyzed their correlation. For this, we needed to define thresholds for differential flux rates and differential gene expression. We applied three thresholds for differential gene expression: a minimal fold change between the conditions of 1.25, a minimal mean logCPM value of 2, and a maximum false discovery rate (FDR) corrected P-value of 0.05. In accordance with Zelezniak *et al*. [26] for enzymes consisting of multiple subunits, we used the fold change value for the subunit with the least fold change and for multiple isoenzymes, catalyzing the same reaction, we used the mean of the isoenzymes’ fold changes. Positive correlations between mRNA and protein abundances have been shown in several studies, e.g. [26–28], giving rise to a positive relation of mRNA and maximal enzymatic turnover rate.

To identify reactions with differential flux rates, we first combined fluxes that use the same set of enzymes (same isoenzymes and subunits, according to gene-protein-reaction associations [14, 29]) by applying the sum of absolute flux rates. In this way, reactions that differ only in cofactor usage are lumped together reducing variability of the individual fluxes. We applied two criteria on the flux sums to identify differential fluxes: The threshold for the minimal difference between the respective condition and the anaerobic control was set to 0.25 $\frac{\mathrm{mmol}}{h\cdot \mathrm{gDCW}}$. The second criterion for differential flux rate was non-overlapping intervals in flux variability analysis (FVA).

**Fig 2:**
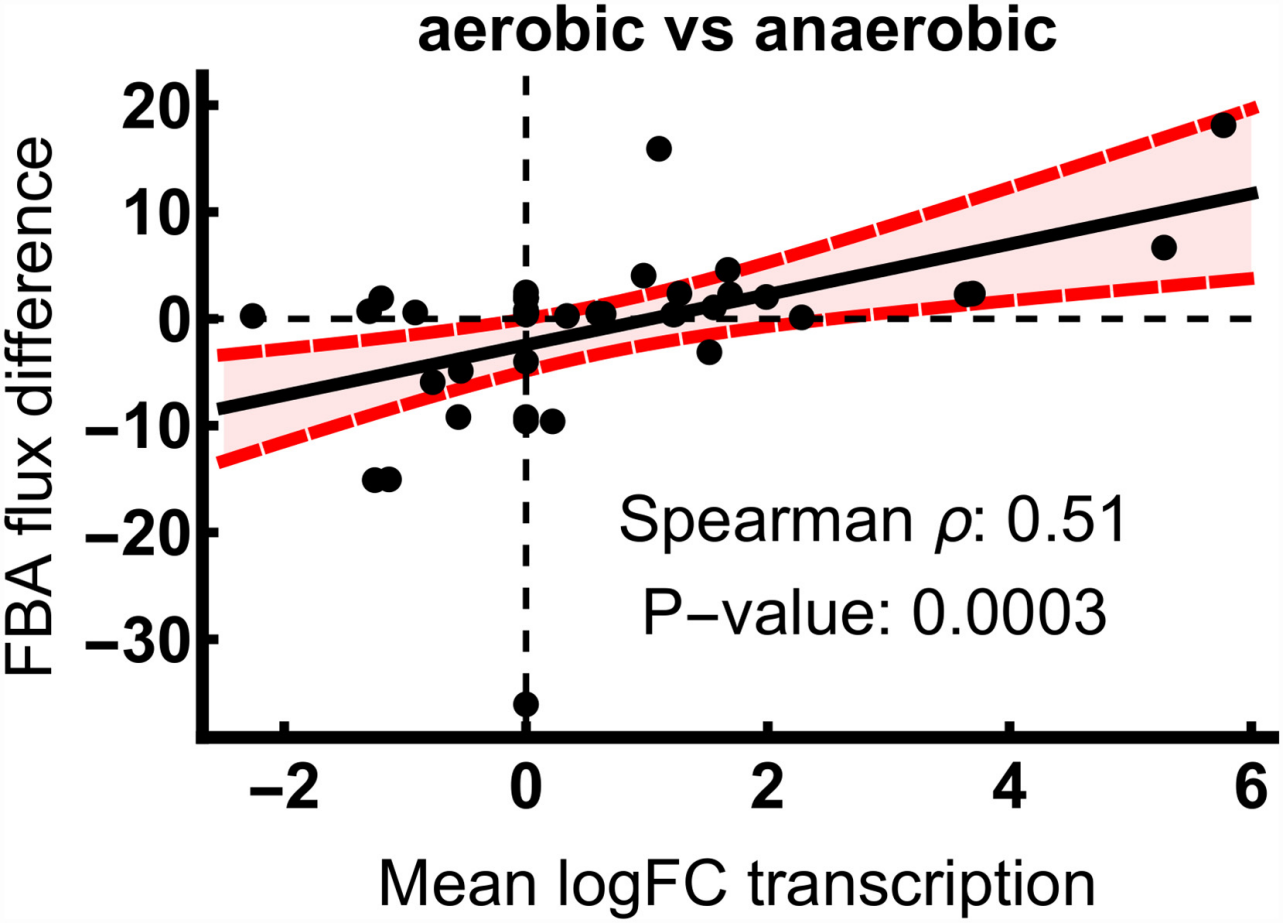
Correlation of transcriptional fold changes with FBA flux rate differences. The difference between the sums of absolute FBA fluxes with identical enzyme composition between anaerobic and aerobic conditions is plotted against the logFC of the associated transcripts. Spearman rank coefficient and P-values are indicated. Linear regression is shown as black line with 95% confidence bands in red.

Fig 2 shows the correlation of flux rate and transcriptome difference with a linear regression. The aerobic steady state expression data correlate well with FBA flux differences with a Spearman rank coefficient of 0.51 and a P-value of 0.003. The linear regression exerts a positive slope, indicating that the relation is also positive. In the following, we want to explore whether this relation appears between a dynamic FBA simulation and transient expression data, as well.

### Method development

As pointed out, to our best knowledge, none of the previously published methods is able to map fast dynamic gene expression data to dynamically changing fluxes. Short-term data of the aerobic shift [5] suggest that fluxes through main aerobic pathways, such as the ETC, are initially constrained by enzyme availability, assuming a correlation between gene expression and fluxes. In order to model the dynamically changing fluxes, we conceived a demand-directed dynamic FBA (dddFBA) that integrates a simulation of dynamic gene expression with enzyme kinetic parameters. The method is based on dFBA with SOA [16], where the fluxes are optimized to present conditions only.

In order to capture gene expression dynamics, balance equations for selected mRNA and proteins are set up.

$$\begin{array}{c} \frac{d\;[mRNA_{i}]}{d\;t}=s_{b,i}+R_{i}\left(t\right)\cdot s_{a,i}-\left(\mu +\gamma_{i}\right)\cdot \left[mRNA_{i}\right]\left(t\right) \end{array}$$ (1)

This equation incorporates a basal transcription rate, *s*_*b*,*i*_, reflecting constitutive expression, an activated transcription rate, *s*_*a*,*i*_, that is switched *on* or *off* depending on the regulatory signal *R*_*i*_(*t*) ∈ {0,1}, dilution with the growth rate *μ*, and a gene specific degradation rate, *γ*_*i*_, adopted from Bernstein *et al*. [30].

The value of the regulatory signal determines whether additional transcription is active or not and is directly responsive to the demand for the respective metabolic flux:

$$\begin{array}{c} R_{i}\left(t\right)=\left\{\begin{array}{cc} 0 & j_{i}\left(t\right)<\theta \cdot b_{i}\left(t\right)\\ 1 & j_{i}\left(t\right)\;⩾\;\theta \cdot b_{i}\left(t\right) \end{array}\right. \end{array}$$ (2)

The regulatory threshold parameter, *θ*, which decides whether a demand for the flux is present (defined as the proportion of flux *j*_*i*_(*t*) to flux bound *b*_*i*_(*t*) that triggers regulation) needs to be chosen between 0 and 1. We chose the value 0.6 reflecting that most enzymes are more than half-maximally occupied by their respective substrates (i.e. substrate concentrations are often slightly higher than K_M_ values, [31]). This choice resulted in good agreement between simulation and measurement of gene expression.

The protein concentration [*P*_*i*_] is modeled in a second ordinary differential equation (ODE, Eq 3). Degradation of proteins in *E*. *coli* is negligible on this time scale and so only the dilution term appears in the equation [32].

$$\begin{array}{c} \frac{d\;[P_{i}]}{d\;t}=s_{P,i}\cdot \left[mRNA_{i}\right]\left(t\right)-\mu \cdot \left[P_{i}\right]\left(t\right) \end{array}$$ (3)

$$\begin{array}{c} s_{P,i}=\frac{\tau }{\Lambda_{i}} \end{array}$$ (4)

We assume the parameter for the protein synthesis rate, *s*_*P*,*i*_, to be inversely proportional to the length of the respective gene, Λ_*i*_ (identified using gene-protein-reaction associations), since ribosomes have to travel along the distance of the gene with limited velocity to produce a polypeptide. The proportionality constant, *τ*, cannot be derived from literature values, because mRNA levels are given in the normalized unit RPKM. The value of *τ* may therefore to be chosen to fit the data. In our case it was taken to be equal to 0.6 $\frac{\mathrm{bp}\cdot \mathrm{mmol}}{h\cdot \mathrm{RPKM}\cdot \mathrm{gDCW}}$ (Eq 4). Finally, the upper bounds *b*_*i*_(*t*) for the respective enzyme fluxes result from

$$\begin{array}{c} b_{i}\left(t\right)=k_{cat,i}\cdot \left[P_{i}\right]\left(t\right) \end{array}$$ (5)

The required parameters were either estimated from transitional gene expression data (*s*_*b*,*i*_, *s*_*a*,*i*_), or adopted from the literature (*γ*_*i*_, *k*_*cat*,*i*_, Table 1).

**Table 1:** Parameters for dddFBA modeling.

reaction	gene	gene length	s_b_ $\left[\frac{\mathrm{RPKM}}{\min}\right]$	s_a_ $\left[\frac{\mathrm{RPKM}}{\min}\right]$	γ [*min^-1^*][30]	s_P_ $\left[\frac{\text{nmol}}{\text{min }\;\text{RPKM }\;\text{gDCW}}\right]$	k_cat_ [*s^-1^*]	Ref.
CYTBD	*cydA*	1569	116.3	759.1 ± 311.1	0.187	10.16	11.7	[33]
CYTBO	*cyoB*	1992	2.4	610.9 ± 88.9	0.210	8.07	341	[34]
NADH5	*ndh*	1305	5.9	504.6 ± 106.8	0.210	12.10	15.8	[35]
NADH16	*nuoG*	2727	25.1	30.0 ± 6.9	10.68	5.95	100	[36]
AKGDH	*sucA*	2802	12.1	143.1 ± 25.1	11.76	4.55	49	[37]
PDH	*aceE*	2664	48.1	2334.0 ± 373.2	7.5	3.79	21.9	[38]

*γ* and *k*_*cat*_ values are adopted from the indicated literature. *s*_*b*_ and *s*_*P*_ are directly calculated from measurement data and gene lengths, respectively. *s*_*a*_ values are estimated from the measurement data, according to Eq 6. 95% confidence intervals are given.

The method contains elements successfully applied to other methodologies. FBA with molecular crowding (FBAwMC [39]) and metabolic modeling with enzyme kinetics (MOMENT [40]) rely on enzyme turnover numbers and restriction arises from limited space of the cytosol available for proteins. These models successfully predict growth rates in multiple media compositions. Similarly, FBA with membrane economics (FBA-ME) limits the available space for membrane-bound proteins and correctly predicts overflow metabolism [41]. E-FLUX limits upper flux bounds according to enzyme capacity that is directly deduced from expression data [24]. This method requires multiple training data that scale the maximal estimated flux to the range of expression data and deduces relative flux bounds from this scaling. Finally, in regulatory FBA (rFBA [17–19]) a Boolean regulatory network determines whether fluxes are allowed or not. This method describes the successive uptake of different carbon sources.

Opposed to these methods we explicitly take gene transcription and translation into account. We do not use measured gene expression values directly to administer bounds to fluxes. Also, we do not impose a regulatory model to determine gene availability but regarded a high flux in relation to its respective bound as a measure for demand for the corresponding enzyme which triggers expression.

Our method takes on the idea from rFBA of switching genes *on* and *off*, but we apply this to transcription instead of enzyme availability, so that continuous flux bounds are obtained. Furthermore, dddFBA does not need a Boolean regulatory model for gene expression, but relies on the flux demand as the regulatory signal. The size of the protein is a critical parameter determining the readiness of availability of a protein. We considered this by normalization of the translation constant *τ* with the respective gene length. For scaling, enzyme kinetic parameters are applied which limit only the upper bound. In contrast to FBAwMC, MOMENT, or FBA-ME, enzyme production is intrinsically limited by defined synthesis and degradation parameters as well as dilution. Expressed mRNA and protein can be regarded as accumulating compounds. In this way, dynamic effects, such as transient expression, arise.

### Simulation

**Fig 3:**
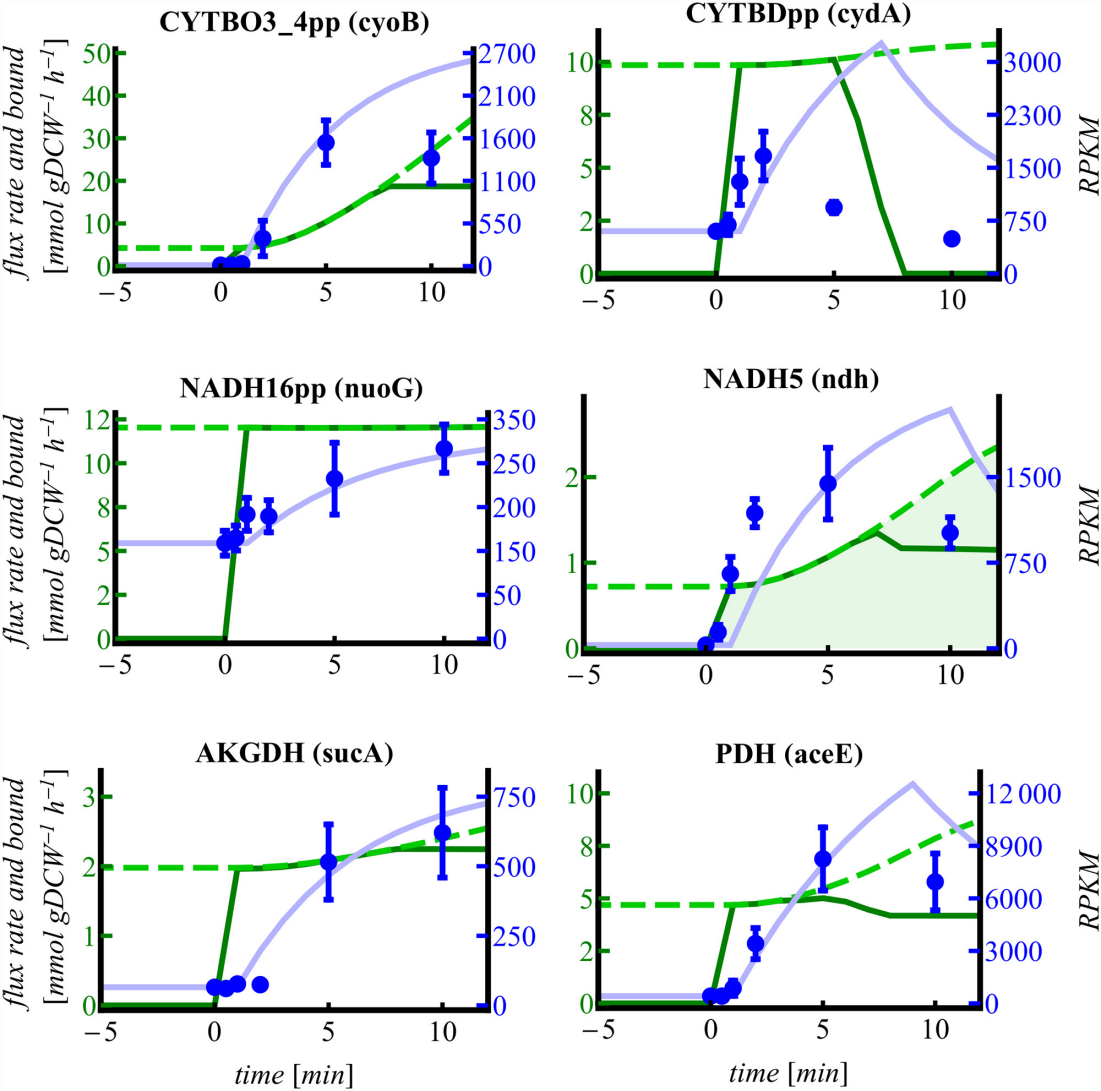
Fluxes of balanced genes in dddFBA. Fluxes are depicted as green lines, upper flux bounds as dashed light green lines, flux variability as shadowed areas and correspond to the left axis; measured mRNA expression as blue dots with standard deviations, and simulated mRNA expression in light blue correspond to the right axis. 0 min denote the onset of aeration.

Simulation of mRNA and protein expression was applied to the reactions and parameters indicated in Table 1. Fig 3 illustrates the short-time upregulation of the MLE reactions CYTBDpp and NADH5 simulated by dddFBA with maximization of growth rate as the objective function. Measured gene expression is qualitatively resembled by the simulation. Expression of MLE genes in the simulation represents a temporary demand for the respective reaction flux.

For FVA, the second optimization of pFBA (minimization of total flux) was omitted and each flux was minimized and maximized keeping the optimal growth rate constant. Flux variability is indicated in Fig 3 by shadowing.

The reaction CYTBDpp (in the following referred to as *cyd*) is MLE compared to CYTBO3_4pp (in the following referred to as *cyo*) as it translocates less protons across the plasma membrane (Fig 1). However, basal anaerobic expression of *cyo* is very low despite its higher aerobic efficiency. The flux through *cyo* reaches its boundary immediately after aeration and expression is turned on. As long as the *cyo* flux is constrained *cyd* is additionally expressed. Furthermore, *cyd* is expressed higher during anaerobic steady state which is probably due to its higher oxygen affinity enabling it to capture even traces of oxygen [42–44]. When *cyo* reaches its final flux value the *cyd* flux drops to zero and *cyd* expression decreases, accordingly. The fluxes of both *cyo* and *cyd* are not variable indicating that the given flux distributions represent the metabolically and enzymatically most efficient solutions.

NADH5 (referred to as *ndh*) is MLE with respect to NADH16pp (referred to as *nuo*) which translocates protons across the plasma membrane (Fig 1). In contrast to the other MLE gene *cyd*, *ndh* is not transcribed anaerobically. The more efficient *nuo* reaction stays constrained throughout the simulation. Despite its very low anaerobic expression, *ndh* is readily available due to its small size. It consists of a single subunit only, whereas *nuo* is a bulky complex consisting of 13 different subunits. Flux through *ndh* is required for an enzymatically efficient solution throughout the simulation causing an overall efficiency loss.

Variability of *ndh* is high and ranges from zero to the upper flux bound indicating that other solutions exist that are metabolically equally efficient. Alternative pathways that reoxidize NADH are listed in Table 2.

**Table 2:** Alternative pathways for reoxidation of NADH.

reaction name	reaction
**malate dependent way**	
MDH (reverse)	malate + NAD^+^ ⇌ oxaloacetate + NADH + H^+^
MDH2	malate + UQ ⇀ oxaloacetate + UQH_2_
**lactate dependent way**	
LDH_D (reverse)	lactate + NAD^+^ ⇌ pyruvate + NADH + H^+^
LDH_D2	lactate + UQ ⇀ pyruvate + UQH_2_
**succinate dependent way**	
MDH (reverse)	malate + NAD^+^ ⇌ oxaloacetate + NADH + H^+^
MDH3	malate + MQ ⇀ oxaloacetate + MQH_2_
FRD2	fumarate + MQH_2_ ⇀ succinate + MQ
SUCDi	succinate + UQ ⇀ fumarate + UQH_2_

**Fig 4:**
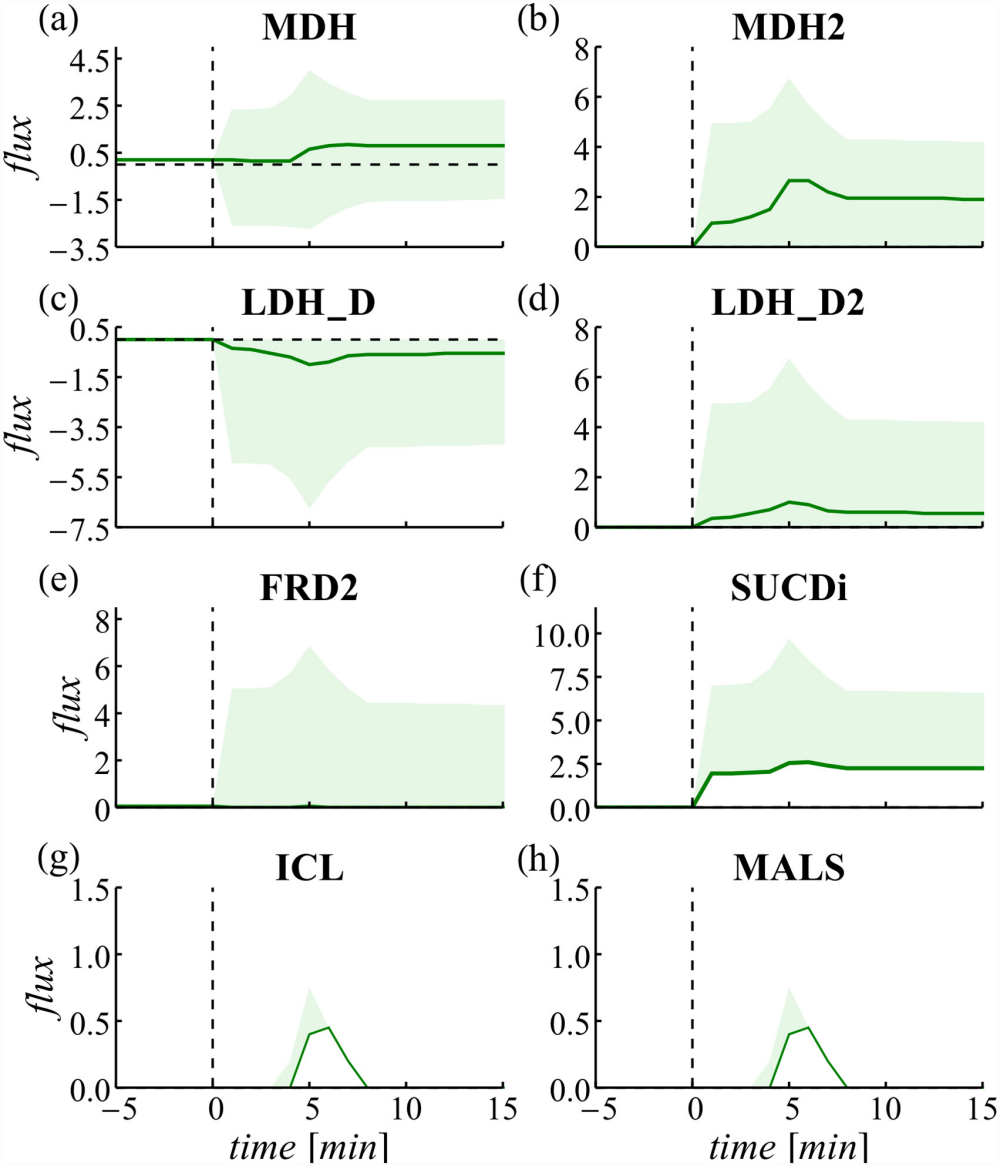
Fluxes of unbalanced reactions in dddFBA. The pFBA solution with minimized squared fluxes is given in black. The shadowed area indicates flux variability.

**Fig 5:**
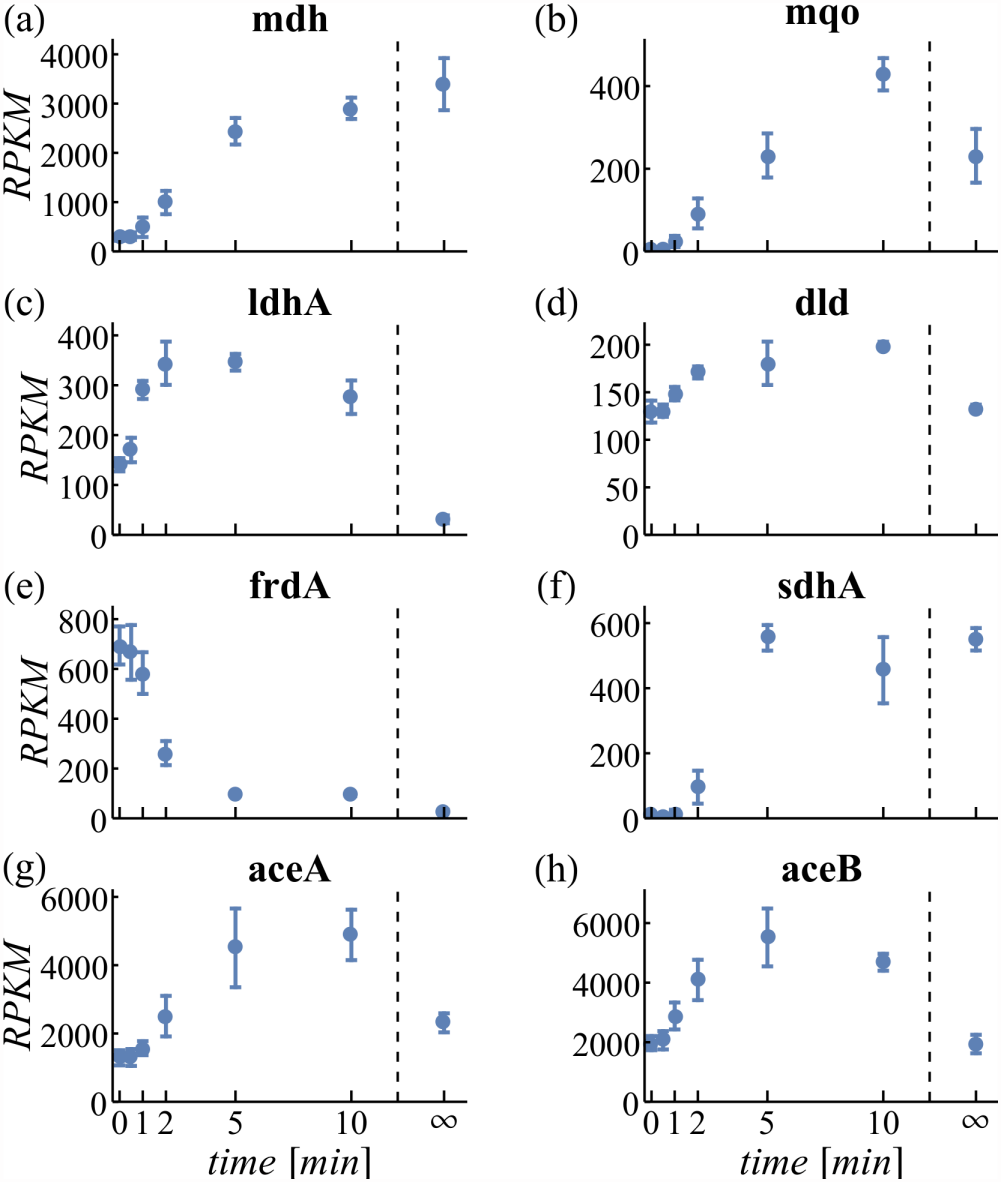
Expression of unbalanced genes. RPKM values of the indicated genes are given with standard deviation. ∞ denotes the aerobic steady-state expression level.

The malate dependent way overlaps with the TCA cycle. MDH transfers electrons from malate to NAD^+^ yielding oxaloacetate and NADH. However, as *nuo* is limited and unable to efficiently reoxidize all NADH generated, the MLE reaction MDH2 assists. This reaction skips NADH and directly transfers the electrons from malate to ubiquinone. However, under *nuo* constrained conditions, MDH2 becomes enzymatically more efficient as it skips the second reaction. This explains the relatively high flux through MDH2 compared to MDH and the high variability of both reactions (Fig 4a and 4b). Expression of both genes, *mdh* catalyzing MDH and *mqo* catalyzing MDH2 increase after oxygenation indicating the demand for both enzymes (Fig 5a and 5b).

Fluxes through LDH_D and LDH_D2 constitute an ELE alternative pathway for NADH reoxidation (c.f. Table 2). Variation for these pathways is high and the pFBA solution predicts a much lower flux usage because of the increased enzyme demand. Still, a slight overexpression of both corresponding genes could indicate a demand for this functionality (Fig 5c and 5d).

Flux through the succinate dependent pathway is not simulated since both, FRD2 and SUCDi, operate at their respective minima (Fig 4e and 4f). Expression of the characteristic protein (encoded by the *frd* operon, Fig 5e) decreases, substantiating the estimated absence of this pathway.

Both C-catabolizing reactions AKGDH and PDH are required for aerobic growth (Fig 3). Their flux is initially constrained resulting in activated transcription which is in good qualitative agreement with the measured data. PDH which forms the connector between glycolysis and the TCA cycle is released earlier from the upper bound constraint resulting in elevated flux through the TCA cycle that is still constrained (by AKGDH). This imbalance is resolved by the temporary utilization of the glyoxylate shunt reactions ICL and MALS (Fig 4g and 4h). The glyoxylate shunt omits several energy conserving steps of the TCA and thus can be perceived as MLE. Transient overexpression of both glyoxylate shunt genes, *aceAB* (Fig 5g and 5h), supports the simulation result.

### Parameter sensitivity

For the presented method parameter sensitivity was analyzed qualitatively by parameter variation. Results of variations of the global parameters translation constant *τ* (Eq 4) and regulatory threshold *θ* (Eq 2, S1 Fig) as well as of the enzyme turnover numbers of CYTBO3_4pp and AKGDH (S2 Fig) are illustrated.

*θ* is varied between the columns of S1 Fig from 0.1 over 0.6 (the value applied in Figs 3 and 4) to 0.9. The observed fluxes prove to be insensitive towards *θ*, only the duration of the expression is influenced *θ* (e.g. expression of *cyo*, encoding the CYTBO3_4pp enzyme, ceases earlier with increasing *θ*, S1a–S1c Fig). *τ* is varied between the rows of S1 Fig. As this parameter determines the height of the upper bound, it influences both, expressions and fluxes. At the intermediate *θ* of 0.6 and a low *τ* of 0.3 $\frac{\mathrm{bp}\cdot \mathrm{mmol}}{h\cdot \mathrm{RPKM}\cdot \mathrm{gDCW}}$, flux through the MLE reaction NADH5 is bounded until it reaches its steady-state value after approximately 35 min (S1b Fig). The initial upper bounds increase linearly with the translation constant and so do the fluxes through the more efficient enzymes. At a *τ* of 0.6 $\frac{\mathrm{bp}\cdot \mathrm{mmol}}{h\cdot \mathrm{RPKM}\cdot \mathrm{gDCW}}$, NADH5 is only bounded until 8 min, whereas no demand for this flux exists any more at a *τ* of 0.9 $\frac{\mathrm{bp}\cdot \mathrm{mmol}}{h\cdot \mathrm{RPKM}\cdot \mathrm{gDCW}}$ (S1e and S1h Fig) since the more efficient NADH16pp is unrestricted.

Similarly, the turnover numbers of the enzymes influence the respective fluxes. We exemplify this by bisection and doubling of the turnover numbers of CYTBO3_4pp and AKGDH leaving both *τ* and *θ* constant at 0.6 (S2 Fig). Again, upper bounds increase linearly with the turnover number, which subsequently decreases the duration of bounded flux and expression (c.f. AKGDH in S2a–S2c Fig). The reduced demand also influences related MLE reactions, such as CYTBDpp whose demand decreases with increasing CYTBO3_4pp turnover (S2b, S2e and S2h Fig).

## Conclusions

Based on the objective function of growth maximization and parsimonious enzyme usage, which are both reasonable assumptions from an evolutionary point of view, and constraints of upper flux bounds reflecting gene expression we simulated mRNA expression using an “on demand” regulatory model to check consistency with measured mRNA. This is in contrast to other methods that incorporate mRNA expression data directly in flux constraints. The presented dddFBA method introduced is able to qualitatively describe the transient overexpression of multiple genes observed during a shift experiment from anaerobic to aerobic conditions. Several overexpressed genes can be classified as MLE or ELE (according to [8]) and thus are not detected by conventional FBA methods. With the introduction of additional constraints arising from gene expression that is directed by the demand for the respective flux we are able to correctly describe transient overexpression. Furthermore, it even enables prediction of temporary fluxes of reactions whose respective enzymes are not explicitly balanced in the model. Qualitatively, the emergence of these temporary fluxes is supported by gene expression data.

Yet, the applicability of this method to other problems is limited. Experimental data are required for parametrization of the model since the estimated transcription rates vary considerably (Table 1).

Still, much can be deduced from this modeling approach: (1), it is reasonable to constrain reaction fluxes with respect to gene expression, (2), limited transcription rates can be conceived as being causal to the (temporary) assignment of less efficient enzymes, (3), constraints on several key enzymes suffice to predict transient behavior of other reactions, as well, and (4), modeling regulation of gene expression in an “on demand” fashion in a pFBA framework with growth optimization results in a realistic gene expression pattern.

A number of simplifications had to be taken to keep the model feasible. We did not account for posttranscriptional or posttranslational modifications that might restrain enzyme activity. Metabolic regulation can be another source for impaired enzyme functioning. However, by applying only a maximal upper bound on the fluxes solutions with inhibitors are still included. Transcription and translation can be seen as transport processes as polymerases and ribosomes migrate along the polynucleotides. The ODEs in Eqs 1 and 3 do not account for that. Instead, translation increases instantaneously with transcription, which is reasonable for *E*. *coli* as both processes occur simultaneously at the same RNA molecule.

In reality, gene expression is not the only limitation applying when optimizing cellular enzyme composition. A multitude of constraints are probably effective, including, (1), membrane occupancy by glucose uptake and ETC proteins [41], (2), molecular crowding of cytoplasm that limits total amounts of translated proteins [39], (3), cells need to find a reasonable tradeoff between growth rate and energetic efficiency, also taking the costs of gene expression into account [6, 45, 46], (4), during a shift of the external conditions, minimal and fast dynamic adjustment of gene expression are required [7], (5), in a dynamic setting, competition for limited RNA polymerases, ribosomes, and tRNAs applies [47], and (6), hampered availability of large protein complexes is also conceivable, e.g. by chaperone efficiency.

These constraints are hard to describe through modeling and even harder to parametrize, especially in a dynamic setting. However, depending on the specific problem and the objective of the modeling, not all limitations need to be taken into account, as demonstrated by the good agreement of the dddFBA simulation with gene expression data.

The data show that MLE enzymes seem to be expressed during the transition from anaerobic to aerobic conditions. Here, we present evidence that this might be due to low availability of the more efficient enzymes. Expression of the MLE enzymes helps to overcome this situation and to increase growth rate, however, this is at the cost of additional gene expression. In a large scale bioreactor with frequently-changing oxygen availability, this finding might be part of the explanation for the observed reductions in growth rate (up to 30%, [48]). Furthermore, as oxygen triggers such a large perturbation, with transiently more than 20% genes of the genome differentially expressed [5], these genes are likely to occupy a good portion of the transcription and translation machinery. These polymerases and ribosomes might then be unavailable for expression of recombinant proteins (up to 94% loss reported, [48]). In the long run, cells will need to adapt to the frequently but nonuniformly changing oxygen supply.

## Materials and Methods

### Data source

We used a dataset of short-time RNA sequencing after an anaerobic to aerobic shift of an *E*. *coli* batch culture available through NCBI’s Gene Expression Omnibus [49] via the GEO Series accession number GSE71562 [5]. The Bioconductor package edgeR [50] was applied for statistical analysis. Read counts were transformed to counts per million (CPM) taking into account the different library sizes. Normalization with the respective gene lengths yields reads per kilobase per million reads (RPKM), which are used for estimating RNA synthesis parameters. A negative binomial model was fitted with the count data and the common and tag-wise dispersions were estimated. Subsequently, a generalized linear model was fitted to the data and p-values were calculated. The resulting p-values were corrected for multiple testing using the FDR method [51]. Genes with p-values of less than 0.05 and absolute fold change greater than 2 were assumed differentially expressed.

### Parameter estimation

From the time course of RPKM values of a gene, synthesis parameters were estimated. This was accomplished by fitting the parameters basal synthesis rate (*s*_*b*,*i*_), activated synthesis rate (*s*_*a*,*i*_, both in $\frac{\mathrm{RPKM}}{\min}$), and the dead time (*T*_*d*,*i*_, in min) of the ordinary differential equation (ODE) Eq 6. Degradation rates (*γ*_*i*_) were adopted from the study of Bernstein *et al*. [30]. The squared brackets denote the concentration of the respective entity.

$$\begin{array}{c} \frac{d\;[mRNA_{i}]}{d\;t}=s_{b,i}+\Theta \left(t-T_{d,i}\right)\cdot s_{a,i}-\left(\mu +\gamma {}_{i}\right)\cdot \left[mRNA_{i}\right]\left(t\right) \end{array}$$ (6)

$$\begin{array}{c} \Theta \left(t\right)=\left\{\begin{array}{cc} 0 & t<0\\ 1 & t\;⩾\;0 \end{array}\right. \end{array}$$ (7)

ODE Eq 6 is analogous to ODE Eq 1, only that the regulatory signal is replaced by the Heaviside function Θ(*t*−*T*_*d*,*i*_). The model (Eqs 6 and 7) incorporates a basal synthesis rate that is assumed to be always active. An additionally activated synthesis rate supports gene expression when the Heaviside function (Eq 7) switches from 0 to 1 after a delay of *T*_*d*,*i*_. Transcribed mRNA_i_ is actively degraded with a certain *γ*_*i*_ and diluted with the growth rate *μ*.

Assuming only basal synthesis for genes that are known to be inactive in an anaerobic environment the initial condition of Eq 6 simplifies to (with Θ(0) = 0)

$$\begin{array}{c} \left[mRNA_{i}\right]\left(0\right)=\frac{s_{b,i}}{\mu +\gamma_{i}}. \end{array}$$ (8)

Inserting RPKM values of a steady state anaerobic culture yields the parameters *s*_*b*,*i*_. The growth rate *μ* was assumed to be 0.26 h^-1^ for anaerobic growth, as measured in preliminary experiments with the same growth conditions. No reasonable measurements of the growth rate could be performed within the 10 min time frame of the original experiment.

Parameter estimations for the yet missing *s*_*a*,*i*_ were performed in *Mathematica* (Wolfram, Oxfordshire, UK) using the function NMinimize and the method SimulatedAnnealing[52] using the least squares estimator.

### Demand-directed dFBA

dddFBA was implemented as described by the Eqs 1 to 5 and integrated in a dynamic pFBA framework, i.e. in each simulation time step, (1) growth rate is maximized, (2) total fluxes are minimized, (3) external metabolite concentrations are updated, and (4) new bounds for uptake reactions, as well as for the balanced reactions are calculated. We used the genome scale model iJO1366 [14] for flux optimization. We applied this technique to the six enzymes listed in Table 1 employing the indicated parameters.

In Eq 4, we assume the parameter for the protein synthesis rate, *s*_*P*,*i*_, to be inversely proportional to the length of the respective gene *i*, since ribosomes have to migrate along the distance of the gene with limited velocity to translate the protein. The value of the translation constant was chosen to be equal to 0.6 $\frac{\mathrm{bp}\cdot \mathrm{mmol}}{h\cdot \mathrm{RPKM}\cdot \mathrm{gDCW}}$ (Eq 4). If a protein is made up of different subunits, the gene coding the largest subunit will be used, because the longest gene is assumed to require the longest time for transcription and translation and so is supposed to be rate limiting for the expression of the holoenzyme. If the enzyme complex is made up of several (n) copies of the longest subunit, the gene length for that subunit becomes an apparent gene length with the interribosomal distance (of 72 bp [53]) added n times. For example, the enzyme AKGDH has a subunit stoichiometry of [(*SucA*)_12_][(*SucB*)_24_][(*Lpd*)_2_]. *sucA* is the longest gene of the enzyme complex with a length of 2802 bp. Since 12 copies of that subunit are required and ribosomes are on average 72 bp apart from each other the apparent gene length is (2802 + 12 · 72)*bp* = 3666 *bp*.

For FVA, the second optimization step was omitted, instead, each indicated reaction was maximized and minimized within the growth optimal solution space [54].

Parameter variations were employed to analyze sensitivity of the respective parameters to fluxes and simulated gene expression. For this, we performed simulations with combinations of the varying parameters translation constant, *θ*, k_cat_(CYTBO3_4pp), and k_cat_(AKGDH).

## Supporting Information

S1 Fig Variation of global parameters. The translation constant is varied between 0.3 and 0.9 (columns); the threshold for regulation is varied between 0.1 and 0.9 (rows).
(PDF)

S2 Fig Variation of k_cat_. The turnover number of CYTBO3_4pp is varied between 170.5 and 682 (columns); the turnover number of AKGDH is varied between 24.5 and 98 (rows).
(PDF)
