# Supplementary material for: Transition of an Anaerobic Escherichia coli Culture to Aerobiosis: Balancing mRNA and Protein Levels in a Demand-Directed Dynamic Flux Balance Analysis
Source: PLoS One. 2016 Jul 6;11(7):e0158711. doi: 10.1371/journal.pone.0158711 (PMC4934858; doi:10.1371/journal.pone.0158711)
Supplement: S1 Fig — The translation constant is varied between 0.3 and 0.9 (columns); the threshold for regulation is varied between 0.1 and 0.9 (rows). (PDF) [file pone.0158711.s001.pdf]

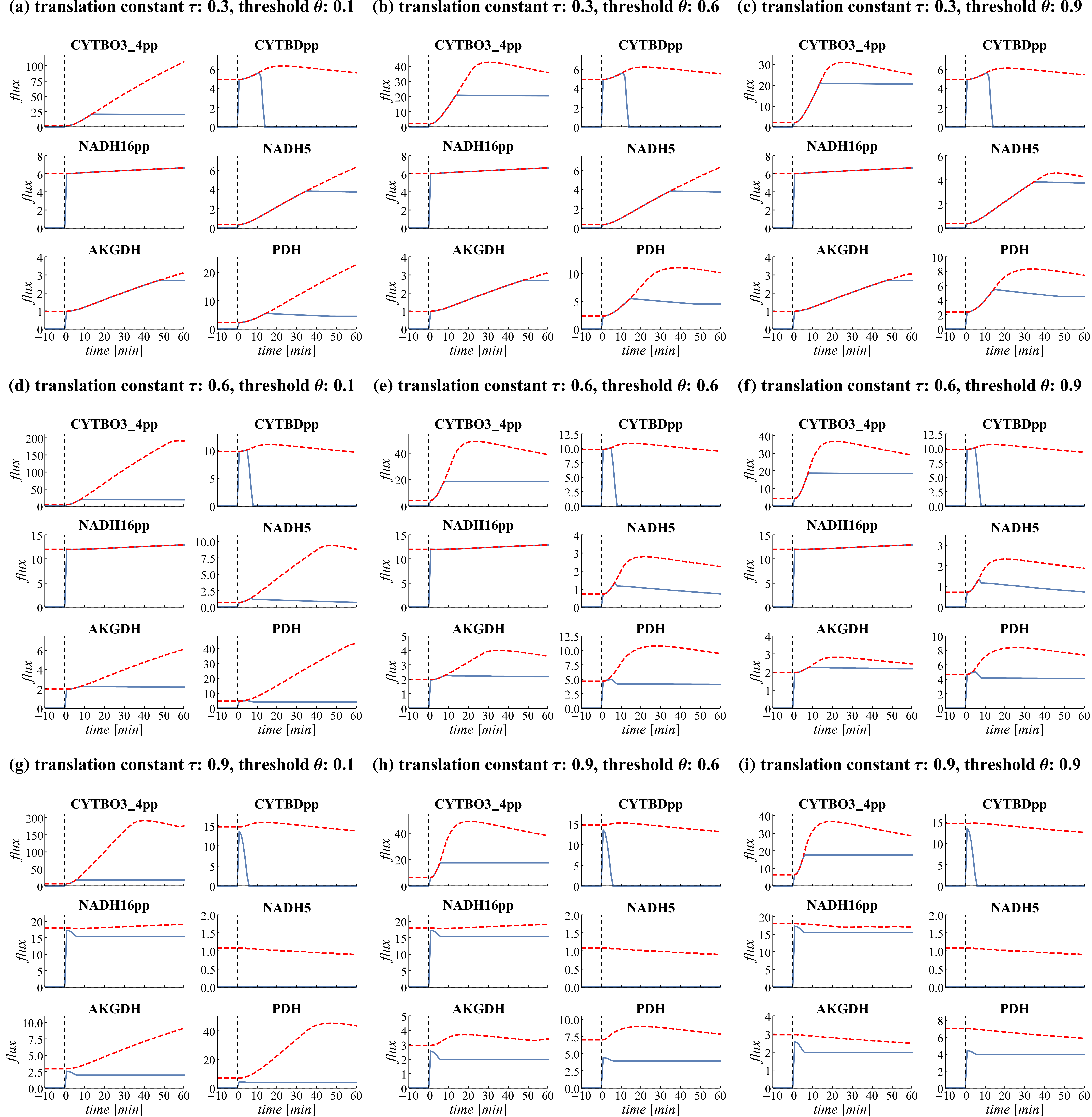

(a) translation constant τ: 0.3, threshold θ: 0.1
(b) translation constant τ: 0.3, threshold θ: 0.6
(c) translation constant τ: 0.3, threshold θ: 0.9
(d) translation constant τ: 0.6, threshold θ: 0.1
(e) translation constant τ: 0.6, threshold θ: 0.6
(f) translation constant τ: 0.6, threshold θ: 0.9
(g) translation constant τ: 0.9, threshold θ: 0.1
(h) translation constant τ: 0.9, threshold θ: 0.6
(i) translation constant τ: 0.9, threshold θ: 0.9
CYTBO3_4pp
CYTBDpp
NADH16pp
NADH5
AKGDH
PDH
flux
time [min]
