# Supplementary material for: Transition of an Anaerobic Escherichia coli Culture to Aerobiosis: Balancing mRNA and Protein Levels in a Demand-Directed Dynamic Flux Balance Analysis
Source: PLoS One. 2016 Jul 6;11(7):e0158711. doi: 10.1371/journal.pone.0158711 (PMC4934858; doi:10.1371/journal.pone.0158711)
Supplement: S2 Fig — The turnover number of CYTBO3_4pp is varied between 170.5 and 682 (columns); the turnover number of AKGDH is varied between 24.5 and 98 (rows). (PDF) [file pone.0158711.s002.pdf]

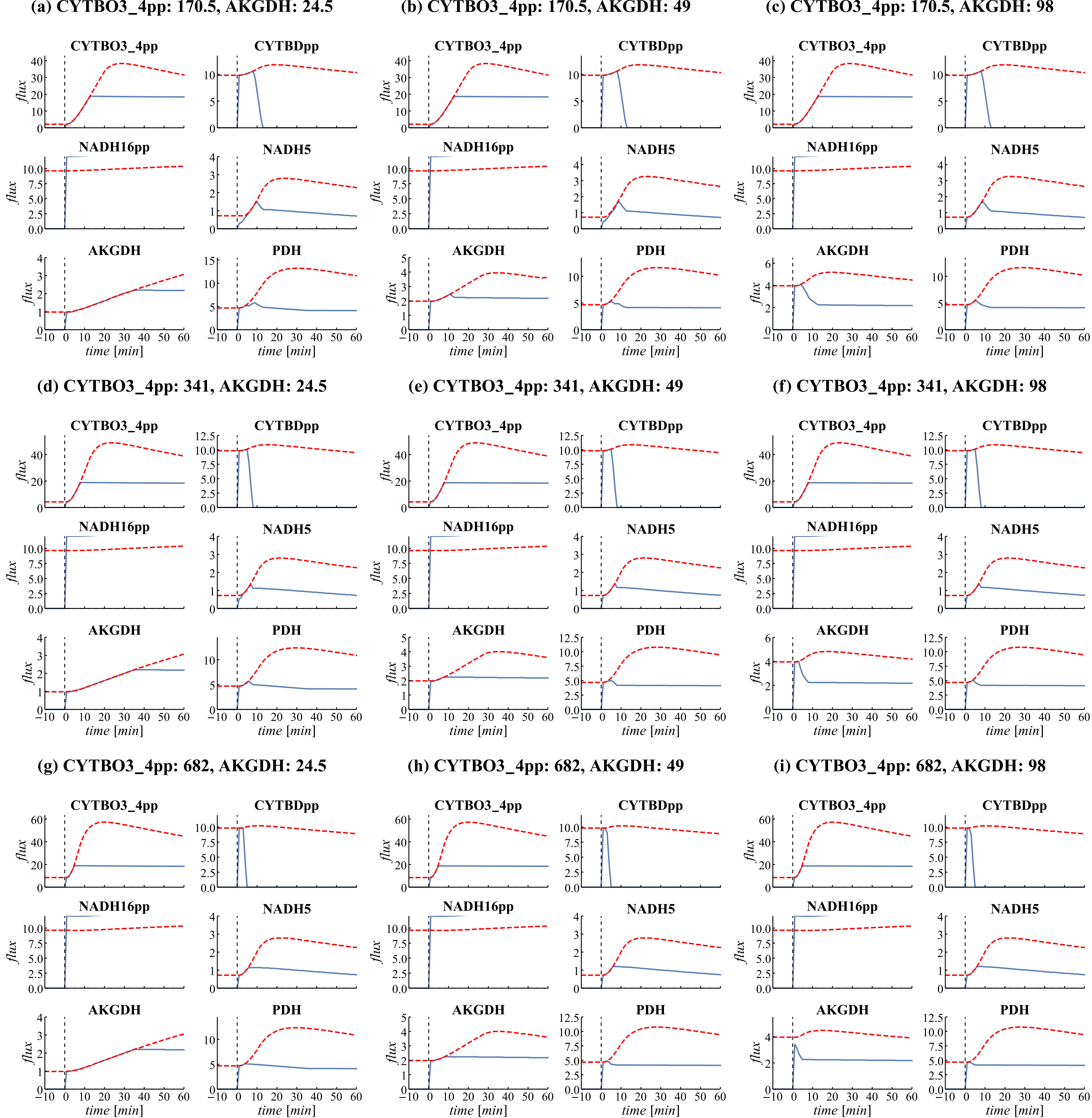
(a) CYTBO3_4pp: 170.5, AKGDH: 24.5
(b) CYTBO3_4pp: 170.5, AKGDH: 49
(c) CYTBO3_4pp: 170.5, AKGDH: 98
(d) CYTBO3_4pp: 341, AKGDH: 24.5
(e) CYTBO3_4pp: 341, AKGDH: 49
(f) CYTBO3_4pp: 341, AKGDH: 98
(g) CYTBO3_4pp: 682, AKGDH: 24.5
(h) CYTBO3_4pp: 682, AKGDH: 49
(i) CYTBO3_4pp: 682, AKGDH: 98
CYTBO3_4pp
CYTBDpp
NADH16pp
NADH5
AKGDH
PDH
flux
time [min]
